# Occurrence of *Campylobacter jejuni* and *Campylobacter coli* in Cattle and Sheep in Northern Spain and Changes in Antimicrobial Resistance in Two Studies 10-years Apart

**DOI:** 10.3390/pathogens8030098

**Published:** 2019-07-08

**Authors:** Medelin Ocejo, Beatriz Oporto, Ana Hurtado

**Affiliations:** NEIKER—Instituto Vasco de Investigación y Desarrollo Agrario, Animal Health Department, Bizkaia Science and Technology Park 812L, 48160 Derio, Bizkaia, Spain

**Keywords:** *Campylobacter jejuni*, *Campylobacter coli*, cattle, sheep, antimicrobial resistance (AMR), Minimum Inhibitory Concentration (MIC), Single Nucleotide Polymorphism (SNP)

## Abstract

A cross-sectional survey was conducted in 2014–2016 in 301 ruminant herds to estimate *C. jejuni* and *C. coli* prevalence, and investigate their susceptibility to antimicrobials. Risk of shedding *C. jejuni* was higher in cattle than sheep (81.2% vs. 45.2%; OR_adj_ = 5.22, *p* < 0.001), whereas risk of shedding *C. coli* was higher in sheep than in cattle (19.1% vs. 11.3%; OR_adj_ = 1.71, *p* = 0.128). Susceptibility to six antimicrobials was determined by broth microdilution using European Committee for Antimicrobial Susceptibility Testing (EUCAST) epidemiological cut-off values. *C. coli* exhibited higher resistance (94.1%, 32/34) than *C. jejuni* (65.1%, 71/109), and resistance was more widespread in isolates from dairy cattle than beef cattle or sheep. Compared to results obtained 10-years earlier (2003–2005) in a similar survey, an increase in fluoroquinolone-resistance was observed in *C. jejuni* from beef cattle (32.0% to 61.9%; OR = 3.45, *p* = 0.020), and a decrease in tetracycline-resistance in *C. jejuni* from dairy cattle (75.0% to 43.2%; OR = 0.25, *p* = 0.026). Resistance to macrolides remained stable at low rates and restricted to *C. coli* from dairy cattle, with all macrolide-resistant *C. coli* showing a pattern of pan-resistance. Presence of the single nucleotide polymorphisms (SNPs) associated to quinolone and macrolide resistance was confirmed in all phenotypically resistant isolates. The increase in fluoroquinolone resistance is worrisome but susceptibility to macrolides is reassuring.

## 1. Introduction

*Campylobacter* is the main cause of food-borne gastroenteritis in industrialized countries and the cause of the most frequently reported zoonosis in the European Union (EU). In 2017, 246,158 confirmed cases of campylobacteriosis in humans were reported in the EU, which accounted to an average notification rate of 64.8 per 100,000 population [1]. In the Basque Country, notification rate in the same year was 104.2/100,000 inhabitants, mainly concentrating among young patients (40.6% in <5 years-old and 20.6% in 5–14 years-old) [2]. The sporadic nature of *Campylobacter* infection causes underreporting and hampers the identification of the infection source [3]. Although poultry is the principal source for human infection, *Campylobacter* is also highly prevalent in ruminants worldwide, and there is increasing evidence that the contribution of ruminant *Campylobacter* to campylobacteriosis in humans is also considerable, with cattle being the second most important reservoir after broilers for *C. jejuni* human infection and sheep the first for *C. coli* infections in humans [4,5]. Contamination of red meat is infrequent [6] and does not seem to play a major risk for human infection, but contaminated raw milk is a frequent vehicle of foodborne infection [7]. In addition, *Campylobacter* from ruminant feces can contaminate water supplies and fresh products via agricultural run-off water [8]. Finally, humans can also get infected by contact with animals. Hence, ruminants are important reservoirs for zoonotic campylobacters and source of contamination for the environment and other animals. Antimicrobial resistance is another subject of concern. Antimicrobial therapy is only recommended in systemic and severe *Campylobacter* infections or in immunocompromised patients. However, the emergence of *Campylobacter* strains resistant to the antimicrobial agents of choice (macrolides for laboratory-confirmed cases and fluoroquinolones for cases of diarrhea) compromises the therapeutic efficacy [9,10]. 

Despite the role of ruminant *Campylobacter* in human campylobacteriosis [5,11,12], the number of studies that estimate *Campylobacter* prevalence in ruminants (dairy cattle, beef cattle and sheep) and investigate their susceptibility to antimicrobials is small compared to the large number of studies carried out in poultry. In a previous study (2003–2006) carried out in ruminants in the Basque Country (Northern Spain), 62.1% of cattle herds and 55.0% of sheep flocks were *Campylobacter*-positive, identifying *C. jejuni* and *C. coli* in 21.8% and 5.9% of the farms, respectively [13]. In that study, all thermotolerant *Campylobacter* species were targeted and only one isolate per positive farm was identified, so that species like *C. hyointestinalis* in cattle or *C. lanienae* in sheep contributed to the high prevalence of the genus *Campylobacter* [14]. A few *C. jejuni* isolates were then characterized for antimicrobial resistance showing high levels of resistance to tetracyclines and quinolones [15]. Now, 10 years later (2014–2016), a similar number of farms was surveyed but targeting only the main zoonotic species and performing a more exhaustive analysis of isolates with the following objectives: (i) To update herd-level prevalence estimates of *C. jejuni* and *C. coli* in ruminant herds in the Basque Country; (ii) to determine the antimicrobial resistance (AMR) profiles of *C. jejuni* and *C. coli*; (iii) to compare antimicrobial resistance in *Campylobacter* isolated from ruminants in two studies carried out 10-years apart; and, (iv) to investigate the potential of a highly discriminatory and rapid method for estimating the true prevalence of *Campylobacter* macrolide-resistance under an apparently low prevalence situation.

## 2. Results

### 2.1. *Campylobacter* Herd Prevalence

**Figure 1:**
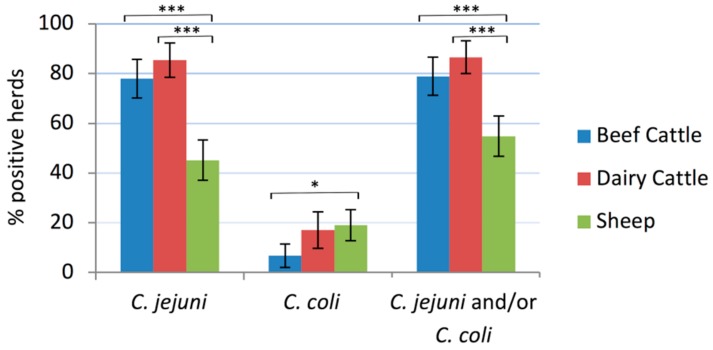
Prevalence of *Campylobacter*-positive herds/flocks in each host. The error bars represent the 95% confidence intervals (*, *p* ≤ 0.05; ***, *p* ≤ 0.001).

The proportion of herds positive to *C. jejuni* and/or *C. coli* was 78.8% (82/104) of beef cattle, 86.6% (71/82) of dairy cattle and 54.8% (63/115) of sheep flocks, the difference in proportions being statistically significant between cattle and sheep (*p* < 0.001) (Figure 1). *C. jejuni* was the most frequently detected species, present in 85.4% of dairy cattle herds, 77.9% of beef cattle herds, and 45.2% of sheep flocks, whereas *C. coli* was found in 17.1% of dairy cattle herds, 6.7% of beef cattle herds and 19.1% of sheep flocks. In 10.0% (30/301) of the tested herds/flocks both *C. jejuni* and *C. coli* were detected. Univariate analysis did not identify any significant explanatory variables associated to *Campylobacter* shedding. Multivariate analysis using host species (cattle vs. sheep) as principal explanatory variable for *C. jejuni* herd prevalence showed that cattle presented significantly higher risk of shedding *C. jejuni* than sheep (OR_adj_ = 5.22 (3.11–8.89), *p* < 0.001), and also when considering the farm system as the principal variable and comparing beef cattle and dairy cattle with sheep separately (dairy cattle vs. sheep: OR_adj_ = 7.07 (3.46–14.43), *p* < 0.001; beef cattle vs. sheep: OR_adj_ = 4.27 (2.36–7.70), *p* < 0.001). No associations were found between *C. jejuni* or *C. coli* shedding and any of the other variables tested. Risk of shedding *C. coli* was non-significantly higher in sheep than in cattle (OR_adj_ = 1.71 (0.86–3.40), *p* = 0.128). However, no differences were found between dairy and beef cattle.

### 2.2. Antimicrobial Susceptibility Tests

**Figure 2:**
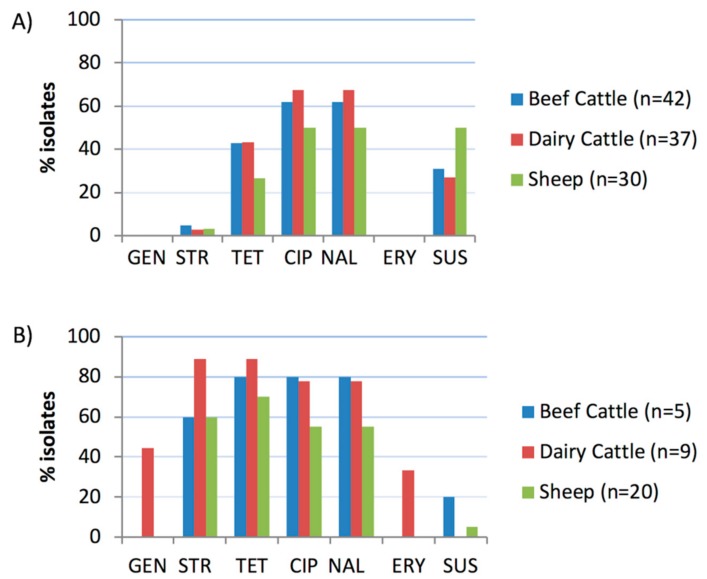
Proportion of isolates resistant to each antimicrobial agent tested: (**A**) *C. jejuni*; (**B**) *C. coli*. GEN, gentamicin; STR, streptomycin; TET, tetracycline; CIP, ciprofloxacin; NAL, nalidixic acid; ERY, erythromycin; SUS, susceptible to all six antimicrobials.

Sensitivities to six antimicrobials (four classes) were determined by broth microdilution for 109 *C. jejuni* isolates and 34 *C. coli* isolates; distributions of minimum inhibitory concentrations (MICs) are shown in Table 1. Isolates susceptible to all antimicrobials tested, accounted for a total of 28.0%. Except for ciprofloxacin and nalidixic acid, resistance against all other antimicrobials was significantly more widespread among *C. coli* than *C. jejuni* isolates (Figure 2) and the magnitude of this difference was higher in isolates from cattle than sheep, and higher in dairy cattle than beef cattle or sheep (Table 2). Overall, 65.1% (71/109) of *C. jejuni* and 94.1% (32/34) of *C. coli* isolates showed resistance to at least one of the six antimicrobial agents tested. *C. coli* exhibited higher resistance to tetracycline (76.5%), streptomycin (67.6%) and quinolones (64.7%), whereas in *C. jejuni* resistance to quinolones (60.6%) was the most common followed by resistance to tetracycline (38.5%) (Table 1). Resistance to quinolones was always present as resistance to both ciprofloxacin and nalidixic acid. Resistance to aminoglycosides varied depending on the antimicrobial agent and the *Campylobacter* species. Thus, whereas all *C. jejuni* were susceptible to gentamicin, 3.7% were resistant to streptomycin; in the case of *C. coli*, resistance to gentamicin was moderate (11.8%) but very high for streptomycin (67.7%). Finally, resistance to erythromycin (macrolide) was low (8.8%) and only detected in *C. coli* isolated from dairy cattle herds. No relationship was found between host species or farm system and resistance against each of the antimicrobial agents tested in neither *C. jejuni* nor *C. coli* isolates. However, MIC values for *C. jejuni* susceptible to gentamicin and streptomycin were higher in sheep than in cattle isolates (*p* = 0.040 and *p* = 0.009, respectively), whereas MICs for *C. coli* resistant to ciprofloxacin and tetracycline were higher in cattle than in sheep isolates (*p* = 0.011 and *p* = 0.029, respectively). 

**Figure 3:**
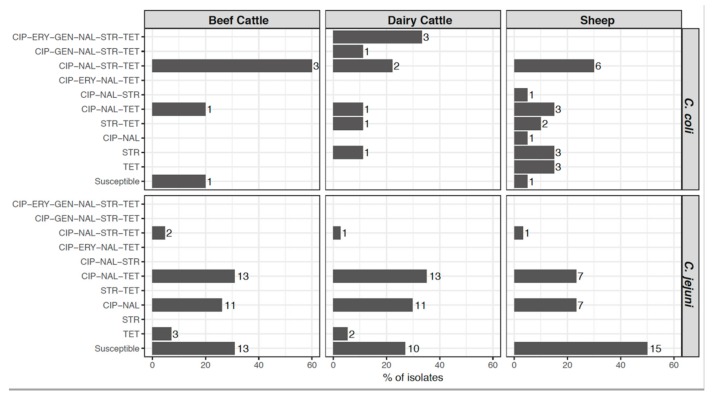
Distribution of antimicrobial resistance (AMR) patterns according to *Campylobacter* species and host. Percentage of each AMR pattern was calculated for each *Campylobacter* species in each host, and numbers beside the bars represent the number of isolates. CIP, ciprofloxacin; ERY, erythromycin; GEN, gentamicin; NAL, nalidixic acid; STR, streptomycin; TET, tetracycline.

**Figure 4:**
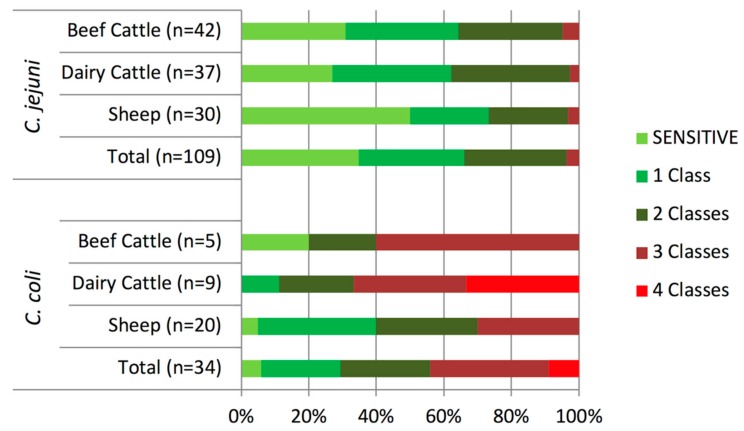
Distribution of isolates resistant to 1–4 classes of antimicrobial agents according to *Campylobacter* species and host.

The distribution of the AMR profiles resulting from the combination of the antimicrobials tested varied according to *Campylobacter* species, but only *C. coli* isolates presented different profiles according to host (Figure 3). Resistance to both quinolones and tetracycline, which was present in 33.9% of *C. jejuni* and 58.8% of *C. coli* isolates, was the combination most commonly found. Multidrug resistance (MDR), defined as resistance to three or more classes of antimicrobial agents, was present in significantly higher proportions (odds ratio (OR) = 20.72, *p* < 0.001) in *C. coli* (44.1%, 15/34) than in *C. jejuni* (3.7%, 4/109) isolates and, albeit at different levels, this trend was observed in each host (Figure 4). Thus, MDR was more likely to occur in *C. coli* than in *C. jejuni*, being the odds ratios much higher in cattle than sheep (OR = 45.60 vs. 12.43), and considerably higher in dairy cattle (OR = 72.00) (Table 2). Among *C. jejuni* isolates, no difference in MDR related to host was detected, and among *C. coli*, MDR was marginally more frequent in isolates from cattle than sheep (OR = 4.20 (0.98–17.95), *p* = 0.053). The most common MDR pattern was to CIP-NAL-STR-TET (Figure 3). All three *C. coli* isolates resistant to erythromycin were also resistant to all other antimicrobials tested. Overall, dairy cattle were the host species that harbored the highest percentage of multidrug resistant *Campylobacter* isolates (15.2%) at similar levels as sheep (14.0%), whereas beef cattle had the lowest (10.6%) (Figure 4). 

### 2.3. Real-Time PCR Discrimination of Single Nucleotide Polymorphisms (SNPs) Associated to Quinolone and Macrolide Resistance

Sequencing analysis demonstrated the analytical specificity of the newly developed TaqMan assay to detect the point mutation C257T in the *gyr*A gene of quinolone-resistant *C. coli*. The results obtained with the real-time PCR SNP discrimination assays were in fully agreement with the phenotypic antimicrobial sensitivity test results. Thus, all *C. jejuni* (66) and *C. coli* (24) isolates that were phenotypically resistant to ciprofloxacin and nalidixic acid contained the C257T mutation in the *gyr*A gene. However, one *C. coli* isolate from a sheep farm failed to give any hybridization results when analyzed with the *C. coli*-specific *gyr*A SNP detection real-time PCR, but provided a resistant profile when using the *C. jejuni*-specific *gyr*A SNP assay. Sequencing analysis of the *gyr*A gene demonstrated that this isolate had a typical *C. jejuni gyr*A allele. The SNP mutation associated to resistance to macrolides (A2075G in the 23S rRNA genes) was found in the only three *C. coli* isolates that were phenotypically resistant to erythromycin. In all three isolates, MIC values were far above the highest concentration tested (>128 mg/L) and therefore far higher than the MIC epidemiological cut-off value. 

### 2.4. Isolation of Macrolide-Resistant *Campylobacter* Using a PCR-Based Screening Method Followed by Selective Isolation

Macrolide-resistant *Campylobacter* was only isolated from one dairy cattle herd when directly picking colonies from the CASA^®^ plate without selective isolation. When DNA extracted from CASA^®^ cultures that tested *C. jejuni* and/or *C. coli*-positive were screened by real-time PCR for the macrolide-resistance associated SNP, another three dairy cattle herds tested positive, and in two of them erythromycin-resistant *C. coli* strains were confirmed after selective isolation in erythromycin-containing media; in the third herd no isolates could be recovered. Hence, prevalence of dairy cattle shedding macrolide-resistant *Campylobacter* was 3.7% (3/82) of herds. 

### 2.5. Changes in *Campylobacter* Antimicrobial Resistance Profiles in Two Studies Carried Out 10-Years Apart

Comparison of results from this study (2014–2016) and those from the study carried out 10 years earlier (2003–2005) showed a significant increase in the proportion of fluoroquinolone resistance in *C. jejuni* isolates from beef cattle (61.9% in 2014–2016 vs. 32.0% in 2003–2005; OR= 3.45 (1.21–9.83), *p* = 0.020). However, resistance to tetracyclines in *C. jejuni* from dairy cattle decreased from 75.0% to 43.2% (OR = 0.25 (0.08 – 0.85), *p* = 0.026). In *C. coli*, no significant changes were observed in the proportion of isolates resistant to each antimicrobial, but MIC values among isolates susceptible to erythromycin were significantly lower in the present study (*p* < 0.001), particularly in isolates from beef cattle (*p* = 0.034).

### 2.6. *Campylobacter coli* Strain Characterization by Multilocus Sequence Typing (MLST)

MLST analysis of 34 *C. coli* isolates resulted in 13 ST-types, 12 of them belonging to clonal complex (CC)-828 and another (ST-8857) not assigned to any recognized CC, and described in this study for the first time. The most common ST type (ST-827) accounted for 38.2% of the *C. coli* isolates typed, and was the most prevalent type in sheep (11/20 isolates; 55.0%) but not in cattle (2/14; 14.3%). All macrolide resistant isolates belonged to ST-2097, a ST-type associated to the three macrolide-resistant *C. coli* isolates plus another MDR *C. coli* (CIP-NAL-GEN-STR-TET) isolated also from dairy cattle.

## 3. Discussion

This cross-sectional study provided estimates of *C. jejuni* and *C. coli* herd prevalence in dairy cattle, beef cattle and sheep in the Basque Country, along with a collection of representative strains and their corresponding antimicrobial resistance profiles. Results showed a widespread distribution of both zoonotic *Campylobacter* species in ruminants, prevalence being higher than that reported in a study carried out in the same region in 2003–2005 [13], an apparent increase that can most likely be ascribed to changes in methodology rather than reflect a real increment. Still, similarly high *Campylobacter* herd level prevalence has been reported in other studies [16,17,18]. Also consistently with other studies in ruminants [18,19], we found that cattle presented significantly higher risk of shedding *C. jejuni* than sheep, while risk of shedding *C. coli* was non-significantly higher in sheep than in cattle. This situation might reflect differences in the epidemiology of *C. jejuni* and *C. coli*. In this sense, increasing evidence suggests that sources and epidemiology of *C. coli* and *C. jejuni* infections are different. Thus, whereas sheep are associated to only 2.5%–24% of *C. jejuni* human infections [11,12], 41% of human *C. coli* clinical cases were attributed to sheep, a proportion similar to that assigned to chicken (40%) and lower than that attributed to cattle (14%) [5]. The higher prevalence of *C. coli* in sheep flocks as described here might explain the significant contribution of sheep to human infection with *C. coli*. On the other hand, it has been suggested that strains isolated from sheep belonged to genotypes that commonly cause disease in humans. Here, half of the strains from sheep belonged to ST-827, one of the genotypes most frequently found in humans [5]. Our MLST results also confirmed the low diversity of *C. coli* STs of ruminant origin compared to *C. jejuni* [20] or compared to *C. coli* from swine or poultry [5]. All but one belonged to the same clonal complex (CC-828) but still, one novel ST was described here (ST-8857). Interestingly, one *C. coli* isolate from a sheep farm (ST-5380) showed a typical *C. jejuni gyr*A sequence suggestive of genome introgressed from *C. jejuni* as previously described for other *C. coli* strains belonging to CC-828 [21]. 

Regarding AMR, differences in methodology and results interpretation hamper comparisons among studies available in the bibliography. Here, EU harmonized methods were used, MICs were determined by broth microdilution, and European Committee for Antimicrobial Susceptibility Testing (EUCAST) epidemiological cut-off values were used to determine microbiological resistance [22]. In this study, resistance against all antimicrobials tested except quinolones was more widespread in *C. coli* than in *C. jejuni* in all three host species, as was MDR. An overall higher resistance in *C. coli* than *C. jejuni* has already been reported in different hosts and for different antibiotics [16,23,24,25,26]. Overall, the highest resistance levels were found for fluoroquinolones (61.5%) followed by tetracycline (47.6%). This represented an increase in the proportion of *C. jejuni* isolates resistant to fluoroquinolones in beef cattle between the two studies carried out 10-years apart but a decrease in resistance to tetracyclines in dairy cattle. In fact, resistance to tetracycline was much lower than that reported for *C. jejuni* isolated from calves in Spain (83.3%) within the EU survey on AMR in zoonotic and indicator bacteria in 2017 [26]. Although overall sales have decreased in the last years, tetracyclines have been used in livestock for many years and in 2016 still accounted for the largest sales (*ca.* 32% of total sales) in the EU and Spain [27]. This would explain the high levels of tetracycline resistance often reported in *Campylobacter* from food-producing animals [28]. The high frequency of fluoroquinolone resistance observed in this study is worrisome, while susceptibility to macrolides is reassuring. Increased resistance of *Campylobacter* to fluoroquinolones has been reported worldwide [9,29], but the levels of fluoroquinolone resistance in both *C. jejuni* and *C. coli* observed in the present study were higher than those found in ruminants in other EU countries [30,31,32], though lower than resistance rates described for poultry and pigs in Spain [26,33]. Use of fluoroquinolones is much higher in Spain than the European average [27]. In a survey carried out among veterinary clinicians in the region (unpublished results), fluoroquinolones were mentioned to be mostly used for diarrhea and respiratory diseases in cattle, similar to data reported elsewhere for Spain [34]. Although the *gyr*A mutation associated to fluoroquinolone resistance has been described to impose certain fitness burden on *Campylobacter* [35], once fluoroquinolone resistant *Campylobacter* is prevalent it can persist for years even in the absence of antibiotic selection pressure so that a reversal in resistance trend might be difficult to achieve [9]. Resistance to macrolides was significantly lower and only detected in *C. coli* isolated from dairy cattle and at similar levels to those reported elsewhere [16,24,25] and in the study carried out 10-years earlier. This is most probably due to the comparatively infrequent use of macrolides in ruminants and the highest fitness cost associated to resistance [9]. Also reassuring was the fact that MIC values among isolates susceptible to erythromycin were significantly lower in the present study than 10 years before. However, macrolide-resistant isolates from both studies were resistant to high concentrations of erythromycin (>128 mg/L) and in most cases co-resistant to fluoroquinolones, tetracycline and aminoglycosides. This pattern of MDR has been associated to the presence of a transferable chromosomal MDR genomic island (MDRGI) that contains the rRNA methylase *erm*(B) gene [36]. Resistance to aminoglycosides was found in a very small proportion of *C. jejuni* isolates, lower than that reported in the EU for cattle isolates [26]. In *C. coli* resistance to aminoglycosides was higher, reaching similar levels to those reported in isolates from pigs [26]. Thus, combined resistance to critically important antimicrobials was absent in *C. jejuni*, but all *C. coli* isolates resistant to erythromycin were also resistant to fluoroquinolones.

Finally, the PCR-based screening method followed by selective isolation in erythromycin-containing media developed here allowed the isolation of macrolide-resistant *C. coli* in two herds that had tested negative when using non-selective isolation. Results demonstrated the usefulness of the method in providing more reliable estimates of macrolide-resistance than procedures that analyze a single colony per sample and likely underestimate the real prevalence. Under a low prevalence situation, PCR screening followed by selective isolation provides the processability needed to carry out extensive antimicrobial surveillance. Here it was only applied to macrolides, but a similar approach could also be used to screen for *C. jejuni* and *C. coli* quinolone resistance prevalence in regions where expected resistance levels were not as high as those found here. 

## 4. Materials and Methods 

### 4.1. Sampling Design

A cross-sectional survey was carried out to estimate the prevalence of *C. jejuni* and *C. coli* in cattle herds and sheep flocks in the Basque Country (Northern Spain). Cattle included both dairy and beef herds, and sheep were of the Latxa dairy breed. Details on general husbandry systems for beef cattle, dairy cattle and sheep in the region were reported elsewhere [37]. Briefly, while dairy cattle are mostly housed in pens, beef cattle and sheep are managed under a semi-intensive system where animals graze in farmland pastures in spring and part of the summer, and in communal mountain pastures from the middle of July until the end of November, and are housed in winter. In all cases, animals of all ages are raised in the herd (suckler herds), and intensive feedlot systems are not used in the region. The census of beef cattle, dairy cattle and sheep farms was obtained from the Department of Agriculture of the Basque Government. The number of herds to sample was then calculated separately for each animal category for an expected herd prevalence of 50%, a 95% confidence level and an accuracy of 10% using Win Episcope 2.0. A sample size of 25 animals per herd was selected after estimating a within-herd prevalence of 10% and a level of confidence of at least 90% in detecting a positive.

Sampling was carried out throughout the year, and a total of 301 herds (115 dairy sheep, 104 beef and 82 dairy cattle) were visited once between February 2014 and June 2016. Rectal fecal samples from 25 animals randomly selected per herd were collected with a gloved hand, and a 25 g-pool was prepared (1 g per animal per pool) for microbiological analyses. Sample collection was carried out by veterinary clinicians as part of the usual health monitoring procedures performed on farms, strictly following Spanish ethical guidelines and animal welfare regulations (Real Decreto 53/2013). The collection of this material, being considered as routine veterinary practice, did not require the approval of the Ethics Committee for Animal Experimentation. Informed oral consent was obtained from the farm owners at the time of sample collection.

### 4.2. *Campylobacter* Isolation and Identification 

For the isolation of thermophilic *Campylobacter* spp., 25 g of pooled rectal fecal samples were diluted 1/10 in Preston broth, homogenized and incubated for 18±2 h at 42 ℃ for enrichment. Suspensions (0.1 mL) were then subcultured onto a Chromogenic-*Campylobacter* Selective Agar (CASA^®^ Agar, Biomerieux) and incubated at 42 ℃ in a microaerobic atmosphere (5% O_2_, 10% CO_2_, 85% N_2_) for 48–72 h. To confirm the presumptive *Campylobacter* and identify the species present in the pool of feces, DNA was extracted from a loopful of bacterial culture (InstaGene, BioRad, CA, USA) and screened for the presence of *C. jejuni* and *C. coli* in a multiplex real-time PCR (TaqMan^®^ *Campylobacter* Multiplex assay, ThermoFisher Diagnostics). Individual colonies were then tested using the same multiplex real-time PCR to confirm their identity and were stored for further characterization. 

### 4.3. Antimicrobial Resistance: Broth Microdilution Tests and SNP Discrimination by Real-Time PCR

Minimum inhibitory concentrations (MIC) were determined by broth microdilution using Sensititre^®^ MIC Susceptibility Plate (ThermoFisher Scientific, Waltham, MA, USA) containing two-fold serial dilutions of six antimicrobial agents (gentamicin, streptomycin, tetracycline, ciprofloxacin, nalidixic acid and erythromycin) following recommendations by the Commission Decision 2013/652/EU. MIC results were interpreted using epidemiological cut-off values (ECOFF) as developed by the European Committee for Antimicrobial Susceptibility Testing (EUCAST, http://www.eucast.org) to define microbiological resistance.

TaqMan real-time PCR assays were used to detect point mutations associated to macrolide (A2075G mutation in the 23S rRNA genes) and quinolone (C257T in the *gyr*A gene, Thr-86-Ile) resistance. Primers and probes used for *C. jejuni* (macrolide and quinolone) and *C. coli* (macrolide) were as described before [15]. To detect point mutations associated to quinolone resistance (C257T in the *gyr*A gene) in *C. coli*, new primers and probes targeting a 101 bp fragment of the *gyr*A gene of *C. coli* were designed in this study (Table 3). PCR reactions and cycling conditions were as previously described [15]. The analytical specificity of the newly developed TaqMan assay to detect the point mutation C257T in the *gyr*A gene of *C. coli* was confirmed by sequencing analysis of the *gyr*A gene amplicon of control strains generated with the primers described in Table 3.

### 4.4. PCR-Based Screening Method for the Isolation of Macrolide-Resistant *Campylobacter*

In order to increase chances of isolation of macrolide-resistant *Campylobacter* (whose prevalence was expected to be very low), a PCR-based screening method followed by selective isolation in media containing erythromycin was assessed. Thus, DNA was extracted from a loopful of bacterial culture grown in CASA^®^ agar (see Section 4.2) from all samples identified as *C. jejuni* and/or *C. coli*-positive and screened with the macrolide SNP real-time PCR assay as described above but using only the probe that detects the resistant SNP (G in nt 2075 of the 23S rRNA gene). All samples that tested PCR-positive were then selectively isolated in Preston broth supplemented with erythromycin (4mg/L, 18±2 h at 42 ℃), and individual colonies (presumptive macrolide-resistant colonies) were identified to the species level by real-time PCR and MICs were determined as above.

### 4.5. Assessment of Changes in *Campylobacter* Antimicrobial Resistance Profiles in Two Studies Carried Out 10-Years Apart

Antimicrobial resistance profiles were available for a 53 *C. jejuni* isolates from a similar study carried out in the region in 2003–2005 (Oporto et al., 2009). As part of this study, MIC values and presence of point mutation associated to quinolone and macrolide resistance were determined for a further 32 *C. jejuni* and 17 *C. coli* isolates from that previous study. Statistical analyses of the results from both studies were performed as described below (Section 4.7).

### 4.6. *Campylobacter coli* Strain Characterization by Multilocus Sequence Typing (MLST)

*C. coli* DNA was extracted from pure cultures using InstaGene (Bio-Rad, Berkeley, CA, USA) and subjected to multilocus sequence typing (MLST) of seven housekeeping genes (*asp*A, *gln*A, *glt*A, *gly*A, *pgm*, *tkt* and *unc*A) as previously described [39,40]. The sequences obtained were queried against the *Campylobacter* MLST database PubMLST (http://pubmlst.org/campylobacter/), developed by Keith Jolley and Man-Suen Chan and sited at the University of Oxford, for type (ST) and clonal complex (CC) assignation. Novel STs were submitted to the *Campylobacter* MLST database for assignation of new numbers.

### 4.7. Statistical Analysis 

Herd-level prevalences were expressed as the percentage of herds/flocks that tested positive in each farm system out of all herds/flocks that were examined in the respective farm system, with 95% confidence intervals adjusted for the population size, using the software EpiInfo2. Variables selected to check for statistical differences in shedding prevalence of each pathogen (i.e., thermophilic *Campylobacter*, *C. jejuni*, *C. coli*) were categorized as follows: Host species (cattle, sheep); farm system (beef cattle, dairy cattle and sheep); sampling season (spring, summer, autumn, winter), geographical location of the farm (oceanic, continental); and presence of other species in the farm such us cattle, sheep, goats, horses (presence, absence). First, univariate logistic regressions were conducted to explore the unadjusted association between herd positivity and variables. Only significant factors (*p* ≤ 0.20; likelihood-ratio test) were included for further multivariate logistic regression analysis. Test of overall significance (chunk test) was performed to assess any possible effect modifiers that could bias the magnitude of associations, and interactions with a value of *p* > 0.05 were excluded until no significant difference between the full and the reduced models was observed. To identify confounding variables, the measure of association was estimated before and after adjusting for the potential confounder, and variables causing change of ≥ 10% in the estimated measure were retained. Adjusted odds ratios (OR_adj_) were used as the measure of association between positivity and the explanatory variable, and were expressed with their confidence interval at 95% (95% CI).

Simple logistic regression tests were performed to determine associations between resistance against the antimicrobial agents tested and host species (sheep vs. cattle) or farm system (beef cattle, dairy cattle and sheep). The same approach was also used to investigate whether resistance against each antimicrobial agent was more likely to be present on either *C. jejuni* or *C. coli*, stratifying the dataset by host species or farm system. Linear regression analyses were performed to compare log_2_ transformed MIC values among host species and farm system, performing independent tests for susceptible and resistant isolates. Results from this study (2014–2016) and those from the study carried out 10 years earlier (2003–2005) were also compared. Qualitative assessment (resistant or susceptible outcome) was done with logistic regression and quantitative comparisons (log_2_ MIC, mg/L) with linear regression. In all cases, regression analyses were performed separately for *C. jejuni* and *C. coli* and for each specific antimicrobial agent. All analyses were conducted in Stata/IC version 13.1 statistical software (StataCorp LP) and figures were elaborated with Microsoft Excel and with ggplot2 package of R statistical software (v.3.3.2).

## 5. Conclusions

This study showed a widespread distribution of *C. jejuni* and *C. coli* in ruminants, with cattle as the main reservoir of *C. jejuni* and sheep of *C. coli*, thus highlighting the importance of non-poultry reservoirs for *Campylobacter* infection. AMR was significantly more prevalent among *C. coli* than *C. jejuni* isolates, and higher in isolates from dairy cattle than beef cattle or sheep. An increase in fluoroquinolone resistance was observed, highlighting the need to promote prudent use of these antimicrobials. Resistance to macrolides, the antibiotic of choice for the treatment of *Campylobacter*-associated diarrhea (especially in infants), remained stable at low rates and restricted to *C. coli*, which was reassuring. Still, antimicrobial surveillance in campylobacters from livestock is needed for early detection of emerging resistance, and the PCR-based screening method developed here proved to be a valuable tool to achieve the processability required for the analysis of a relatively large number of samples. 

## Figures and Tables

**Table 1:** Microbiological resistance (percentage) and distribution of minimum inhibitory concentrations (MICs) for the 109 *C. jejuni* and 34 *C. coli* isolates.

Antimicrobial Class	Antimicrobial Agent	*Campylobacter* Species	% Resistance				No. of Isolates at the Indicated MIC (mg/L)												
			TOTAL	Beef Cattle	Dairy Cattle	Sheep	0.064	0.125	0.25	0.5	1	2	4	8	16	32	64	128	256
Aminoglycoside	Gentamicin	*C. jejuni*	0.0	0.0	0.0	0.0		3	12	87	7								
		*C. coli*	11.8	0.0	44.4	0.0				15	11	4				4			
Aminoglycoside	Streptomycin	*C. jejuni*	3.7	4.8	2.7	3.3				1	17	84	3			4			
		*C. coli*	67.7	60.0	88.9	60.0						8	3	5	1	17			
Macrolide	Erythromycin	*C. jejuni*	0.0	0.0	0.0	0.0					109								
		*C. coli*	8.8	0.0	33.3	0.0					31								3
Quinolone	Nalidixic acid	*C. jejuni*	60.6	61.9	67.6	50.0						1	31	11			6	60	
		*C. coli*	64.7	80.0	77.8	55.0							3	9			3	19	
(Fluoro)Quinolone	Ciprofloxacin	*C. jejuni*	60.6	61.9	67.6	50.0		43					1	41	24				
		*C. coli*	64.7	80.0	77.8	55.0		11	1					6	9	7			
Tetracycline	Tetracycline	*C. jejuni*	38.5	42.9	43.2	26.7				67			2		2	6	23	9	
		*C. coli*	76.5	80.0	88.9	70.0				8						3	2	21	

White fields denote range of dilutions tested for each antimicrobial agent. MICs above the range are given as the concentration closest to the range. MICs equal to or lower than the lowest concentration tested are given as the lowest tested concentration. Vertical lines indicate European Committee for Antimicrobial Susceptibility Testing (EUCAST) epidemiological cut-off values.

**Table 2:** Odds ratio (OR) of association between resistance to each antimicrobial and *Campylobacter* species for each host, determined with simple logistic regression analysis using *C. jejuni* as reference.

AMR ^a^	Sheep		Cattle		Beef Cattle		Dairy Cattle	
	OR (95% CI)	*p*	OR (95% CI)	*p*	OR (95% CI)	*p*	OR (95% CI)	*p*
CIP/NAL	1.22 (0.39–3.80)	0.729	2.01 (0.52–7.82)	0.312	2.46 (0.25–24.02)	0.438	1.68 (0.30–9.34)	0.553
ERY ^b^	NA		NA		NA		NA	
TET	6.42 (1.83–22.46)	0.004	7.94 (1.67–37.86)	0.005	5.33 (0.55–51.88)	0.149	10.50 (1.19–92.72)	0.034
GEN ^b^	NA		NA		NA		NA	
STR	43.5 (4.89–386.74)	0.001	92.89 (16.62–519.09)	<0.001	30.00 (3.06–294.56)	0.004	288.00 (16.23–5108.64)	<0.001
MDR	12.43 (1.36–113.41)	0.025	46.60 (9.30–223.47)	<0.001	30.00 (3.06–294.56)	0.004	72.00 (6.39–811.79)	0.001

^a^ CIP, ciprofloxacin; NAL, nalidixic acid; ERY, erythromycin; TET, tetracycline; GEN, gentamicin; STR, streptomycin; MDR, multidrug resistance pattern, defined as resistance to three or more classes of antimicrobial agents. ^b^ All *C. jejuni* were susceptible and therefore associations cannot be calculated (NA, non-applicable). *p* < 0.05 are considered significant.

**Table 3:** Primers and probes used to detect point mutations associated to macrolide (A2075G mutation in the 23S rRNA genes) and quinolone (C257T in the *gyr*A gene, Thr-86-Ile) resistance in *C. jejuni* and *C. coli*, and *gyr*A sequencing.

Target	Name	Sequences (5’ → 3’)	C (nM)	Reference
*C. jejuni gyr*A C257T	gyrCj-Fw	GGGTGCTGTTATAGGTCGTTATCA	900	[15]
	gyrCj-Rv	TTGAGCCATTCTAACCAAAGCAT	900	
	Probe-gyrCj-S	HEX–CAT[+G]GAGAT[+A][+C][+A]GC[+A]GTTT–BHQ1	150	
	Probe-gyrCj-R	FAM–CATGGAGATATAGCAGTTT–MGB	150	
*C. coli gyr*A C257T	gyrCc-Fw	GAAGTGCATATAAAAAATCTGCTCGTA	400	This study
	gyrCc-Rv	TGCCATTCTTACTAAGGCATCGT	400	
	Probe-gyrCc-S	FAM–AACAGCAGTATCGCC–MGB	150	
	Probe-gyrCc-R	VIC–AACAGCAATATCG–MGB	150	
23S rRNA A2075G	23S-Fw	CAGTGAAATTGTAGTGGAGGTGAAA	900	[15]
	23S-Rv	TTCTTATCCAAATAGCAGTGTCAAGCT	900	
	Probe-23S-S	HEX–CGGGGTC[+T][+T][+T]CCGTCTTG–BHQ1	100	
	Probe-23S-R	FAM–CGGGGTC[+T][+C][+T]CCGTCTTG–BHQ1	200	
*C. coli gyr*A sequencing	GZgyrACcoli3F	TATGAGCGTTATTATCGGTC	400	[38]
	GZgyrACcoli4R	GTCCATCTACAAGCTCGTTA	400	

Locked nucleic acids (LNA) are indicated by a + symbol and in brackets; SNPs are underlined.
